# Valuable Clues for DCNN-Based Landslide Detection from a Comparative Assessment in the Wenchuan Earthquake Area

**DOI:** 10.3390/s21155191

**Published:** 2021-07-31

**Authors:** Chang Li, Bangjin Yi, Peng Gao, Hui Li, Jixing Sun, Xueye Chen, Cheng Zhong

**Affiliations:** 1 Three Gorges Research Center for Geo-Hazard, Ministry of Education, China University of Geosciences, Wuhan 430074, China; lichang_net@cug.edu.cn; 2 Yunnan Institute of Geological Science, Kuming 650051, China; bangjinyi@sina.com (B.Y.); sunjixing8@gmail.com (J.S.); 3 Department of Earth and Ocean Sciences, University of North Carolina, Wilmington, NC 28403, USA; gaop@mailbox.sc.edu; 4 Department of Geography, University of South Carolina, 709 Bull St., Columbia, SC 29208, USA; 5 School of Earth Sciences, China University of Geosciences, Wuhan 430074, China; rslihui@cug.edu.cn; 6 Key Laboratory of Urban Land Resources Monitoring and Simulation, MNR, Shenzhen 518034, China; xueyers@sina.com

**Keywords:** landslide detection, deep learning, Wenchuan earthquake, ResNet, DenseNet, U-Net

## Abstract

Landslide inventories could provide fundamental data for analyzing the causative factors and deformation mechanisms of landslide events. Considering that it is still hard to detect landslides automatically from remote sensing images, endeavors have been carried out to explore the potential of DCNNs on landslide detection, and obtained better performance than shallow machine learning methods. However, there is often confusion as to which structure, layer number, and sample size are better for a project. To fill this gap, this study conducted a comparative test on typical models for landside detection in the Wenchuan earthquake area, where about 200,000 secondary landslides were available. Multiple structures and layer numbers, including VGG16, VGG19, ResNet50, ResNet101, DenseNet120, DenseNet201, UNet−, UNet+, and ResUNet were investigated with different sample numbers (100, 1000, and 10,000). Results indicate that VGG models have the highest precision (about 0.9) but the lowest recall (below 0.76); ResNet models display the lowest precision (below 0.86) and a high recall (about 0.85); DenseNet models obtain moderate precision (below 0.88) and recall (about 0.8); while UNet+ also achieves moderate precision (0.8) and recall (0.84). Generally, a larger sample set can lead to better performance for VGG, ResNet, and DenseNet, and deeper layers could improve the detection results for ResNet and DenseNet. This study provides valuable clues for designing models’ type, layers, and sample set, based on tests with a large number of samples.

## 1. Introduction

In recent years, geological disasters (collapses, landslides, debris flows, etc.) have remarkably increased, influenced by global climate change, seismic activity, and accelerated urbanization processes. According to the national geological disaster report of China, 409,004 geological disasters (including 293,104 landslides) occurred from 2000 to 2020, resulting in 12,222 deaths or missing persons, and USD 12.3 billion in economic losses [1]. Landslide inventories could provide fundamental data for investigating the causative factors and deformation mechanisms of landslides [2,3,4]. Remote sensing has been widely used in landslide detection and monitoring as well as hazard mapping [5,6], as it can monitor earth surface changes over a large area quickly and cost effective, compared to field investigations [7,8]. Although delineating landslides manually from images has been broadly accepted and employed in practice, it lacks objectivity and efficiency [9,10,11].

Recently, many endeavors have been made for mapping landslides semi-automatically or automatically, including threshold segmentation [12,13], change detection [14,15,16], unsupervised and supervised classification [17,18], object-oriented analysis, and machine learning algorithms [6]. In addition, some novel data sources—such as differential interferometric synthetic aperture radar (DInSAR), hyperspectral images, light detection and ranging (LiDAR), and unmanned aerial vehicles (UAVs)—have been adopted for finding more spatial, temporal, and spectral characteristics of landslides [19,20,21]. However, several facts impose great challenges in landslide detection [6]. First, the spectral, spatial, and temporal characteristics of landslides are often similar to bare slopes, ranks, etc. Second, there may be huge differences between landslides in different geo-environments. Third, landslide elements (such as main scarps, tongues, bodies, etc.) are often characterized by different spectral, spatial, and temporal appearances. Thus far, it is still hard to identify landslides accurately and automatically from complex image scenes.

Recently, deep learning has achieved great success in computer vision, image processing, and other fields [22,23]. Deep convolutional neural networks (DCNNs) have great advantages in understanding complex scene semantics by mining multilevel (low to high) image features independently [24,25]. Recently, several tests on landslide detection with deep learning models have been carried out to explore the potential of DCNNs to identify landslides in complex scenes [26,27]. The authors of [28] found that a DCNN model with small window size achieved better mIoU (78.26%) than typical machine learning algorithms (e.g., random forests, support vector machines, and artificial neural networks). In a study in Cameron Highlands, Malaysia, the authors of [29] discovered that a residual network (ResNet) with feature-level fusion achieved a higher F1 score and mIoU than a one-layer CNN with two deeper counterparts, or a ResNet with layer stacking. In [30], an attention-boosted CNN model was tested, and achieved a high F1 score (96.62%). In [31], a modified U-Net model with ResNet34 blocks was tested, and obtained a better performance than conventional methods, for detecting landslides at a regional scale from Earth observation satellite images. In [32], LandsNet—a cascaded DCNN network—was proposed for landslide detection, and achieved a high F1 score (86.89). In [33], an object-oriented change detection technique based on a trained CNN was presented for landslide detection, and displayed high speed and accuracy (80%). In [34], a Mask R-CNN with pixel-based segmentation was proposed, and obtained good precision (1.00), recall (0.93), and F1 score (0.97). 

Although many deep learning models have been proposed and compared with traditional machine learning algorithms, the comprehensive comparative assessment on them is lacking, which often makes decision-makers confused as to which structure, layer number, and sample size of DCNN are suitable for landslide detection. With this in mind, we conducted a comparative test of typical deep learning models in the Wenchuan earthquake area, where 197,482 seismic secondary landslides were available for model training and validation. In the experiment, multiple structures and layer numbers—including VGG16, VGG19, ResNet50, ResNet101, DenseNet120, DenseNet201, UNet−, UNet+, and ResUNet—were investigated with three sample sets (S100, S1000, and S10000).

## 2. Materials and Methods

### 2.1. Study Area

**Figure 1:**
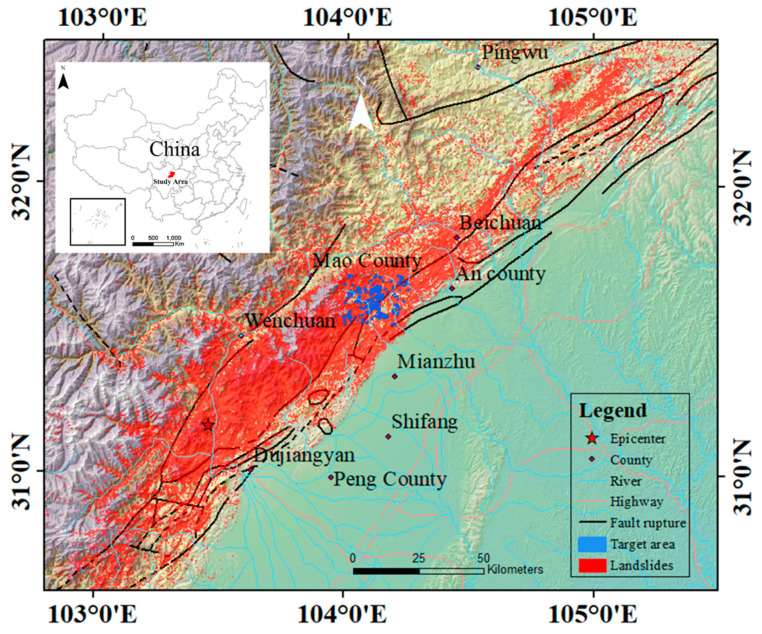
The study area with landslides caused by the 2008 Wenchuan earthquake and fault ruptures. Tests on the blue “target area” as examples of landslide detection.

The Wenchuan earthquake area is located at the conjunction of Sichuan basin and the Tibetan plateau (Figure 1), covering Wenchuan, Maoxian, Beichuan, and eight other counties, covering an area of about 15,000 km^2^. In this area, the Longmenshan Fault Zone—consisting of the Maowen Fault, the Yingxiu–Beichuan Fault, and the Dujiangyan–Anxian fault—is widely known as a seismic belt [35]. Mountains higher than 3000 m dominate in the western part of the area, while low hills and plains occupy the eastern part. The region has a temperate monsoon climate, which is characterized by uneven sunlight, heat, and water distribution. In the area, slope failure occurs easily due to plate activity, steep slopes, loose soil, and extreme precipitation.

Post-earthquake field survey and remote sensing image interpretation showed that 197,481 secondary landslides were triggered by the Wenchuan earthquake [36]. These landslides are distributed over a great area (about 110,000 km^2^), with elevation from 800 m to 4500 m. After the 2008 earthquake, the area exhibited debris flows, reactivations, and remobilizations in the subsequent monsoon periods [37,38]. 

### 2.2. Data and Samples

The landslide inventory used in this study was interactively interpreted from high-resolution images (SPOT, QuickBird, etc.) after the earthquake [35]. A total of 197,482 landslide polygons with location, area, and elevation were available in this inventory. Worldview-2 images from the year 2012 with cloud cover less than 10% were selected for landslide detection. In these images, most landslides were greater than 5 pixels; thus, their spectral and spatial characteristics were clear and detectable. 

Among 197,482 rectangular patches cut out from images according to landslide polygons, 10,000 patches where landslides were clear and not covered were selected as the original sample set, and named S10000. The spatial distribution of original samples was similar to that of the landslides in Figure 1. Furthermore, two smaller sample sets—namely, S100 and S1000, whose sample numbers were 100 and 1000, respectively—were randomly selected from the original sample set. The three training sample sets were used to test the dependency of models on sample numbers

### 2.3. Methods 

**Figure 2:**
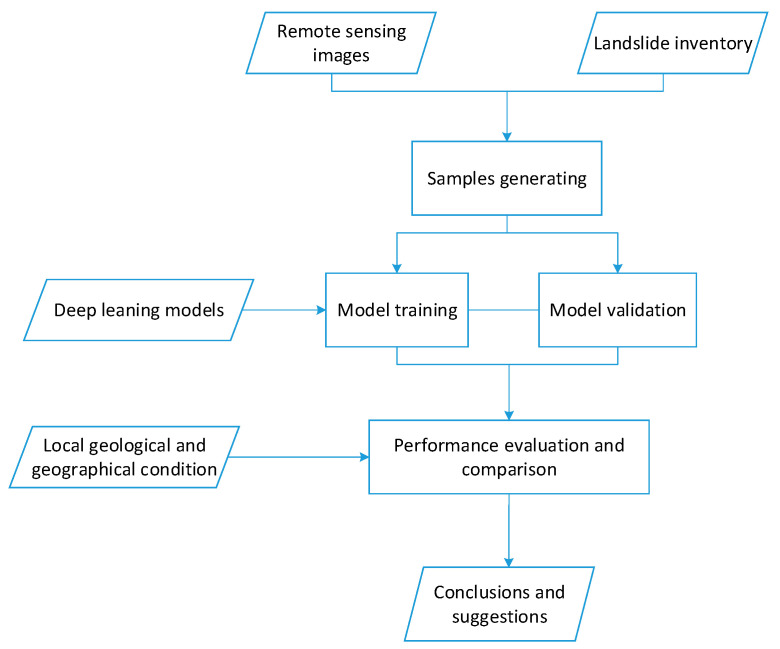
The flowchart of this study.

The flowchart of this study is shown in Figure 2. First, samples were generated from remote sensing images with landslide polygons; then, typical deep leaning models (e.g., VGG neural networks (VGG), residual networks (ResNet), densely connected networks (DenseNet), and U-Net) with different structures and layer numbers were trained and validated with three sample sets (S100, S1000, and S10000), and then their performances were evaluated and compared; finally, suggestions for further study and projects were given. 

#### 2.3.1. VGG Models

**Figure 3:**
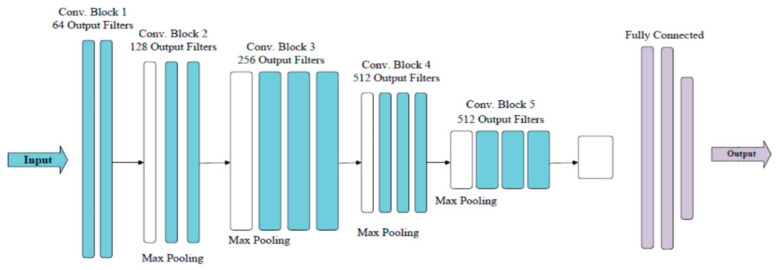
The structure of a VGG model (derived from [39]).

As shown Figure 3, multiple 3 × 3 convolution kernels are involved in a VGG model, as opposed to the larger convolution kernels (11 × 11, 7 × 7, 5 × 5) used in AlexNet [39]. Compared with AlexNet, VGG can learn more complex patterns at a lower cost (fewer parameters), as the depth of the network remarkably increases when adding multiple nonlinear convolution kernels. For instance, in VGG, three 3 × 3 convolution kernels were often used to replace one 7 × 7 convolution kernel from AlexNet. 

Previous studies proved that multiple small convolution kernels could enhance the feature learning ability of the model. It is easy to obtain deeper and more abstract features with a VGG model [25]. Moreover, VGG has strong generalization ability. A trained VGG model could be smoothly used in other images, and achieved good performance. Typical VGG models—VGG16, and VGG19—were tested in this study, as they have shown outstanding performance in many related studies [40]. VGG16 consists of 13 convolutional layers and 3 fully connected layers, while VGG19 has 16 convolutional layers and 3 fully connected layers. 

#### 2.3.2. ResNet Models

**Figure 4:**
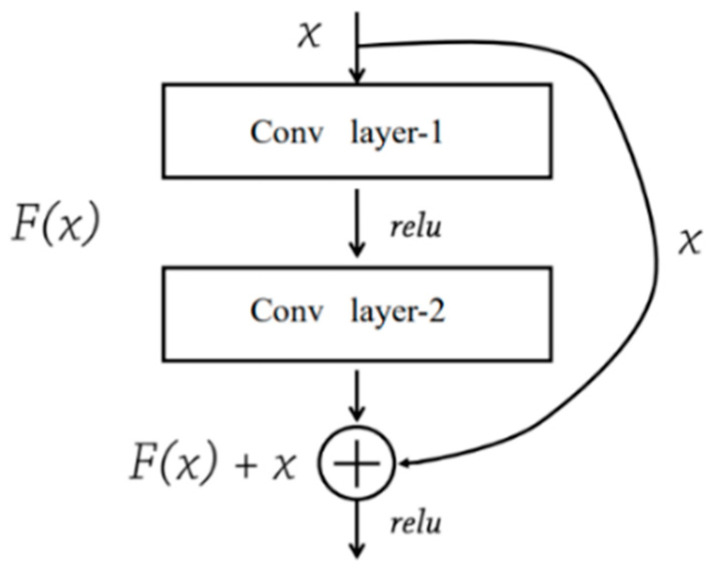
A residual block (derived from [41]).

As shown in Figure 4, a residual block—the basic unit of a ResNet—consists of two convolutional layers and a shortcut connection. Through shortcut connections, residuals can be quickly transferred, and gradient problems can be alleviated. The ResNet ensures that the input information can be completely propagated among all layers by using the “shortcut connection” [41]. ResNet can effectively avoid gradient loss or exploding gradients when its network becomes very deep, which implies that the model may improve landslide detection accuracy by increasing its number of layers. No additional parameter or calculation is needed for adding shortcut connection to the network. Two typical ResNet models—ResNet50, and ResNet101—were tested in this study. 

#### 2.3.3. DenseNet Models

**Figure 5:**
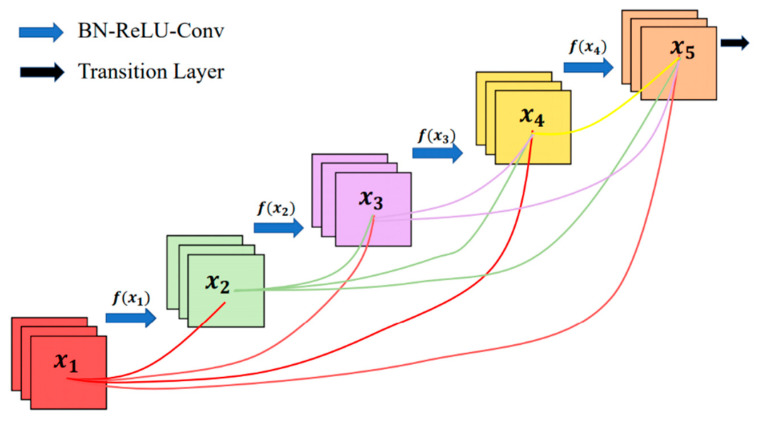
The structure of DenseNet (derived from [42]).

In the DenseNet proposed by [42], all layers connect with one another, in order to ensure that information can be transmitted between layers (as shown in Figure 5). DenseNet consists of dense blocks and transition layers; two adjacent dense blocks are connected by a transition layer, which includes a 1 × 1 convolutional layer and a 2 × 2 average pooling layer. Feature maps and gradients can be effectively transmitted between dense blocks through transition layers. Then, gradient loss can be easily avoided in this model, as each layer can directly get the input information and loss function. 

DenseNet greatly reduces the number of parameters by using a dense block structure. It has a strong ability of anti-overfitting, due to the modified manner of information and gradient transfer. Furthermore, it can be effectively trained even if samples are relatively insufficient, as the connected dense blocks have a regularization effect [43]. Two typical DenseNet models—DenseNet120, and DenseNet201—were tested in this study. 

#### 2.3.4. U-Net models 

**Figure 6:**
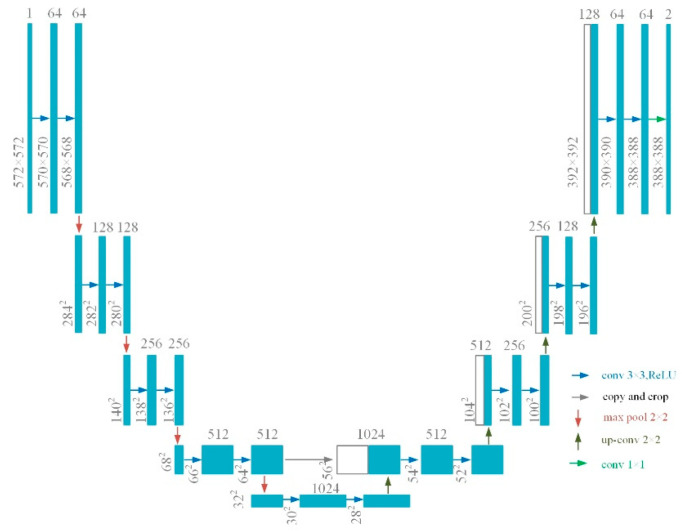
The structure diagram of U-Net.

U-Net was proposed to reduce the loss of feature maps and spatial location information in the pooling process of the fully convolutional networks [44]. In the model, the original image is first transformed to a high-dimensional feature vector through 3x downsampling with a fully connected neural network, and then the vector is restored to a feature map whose resolution is the same as the original image, through 3x upsampling [45] (Figure 6). Each layer of the model has a skip connection, which ensures that features of different layers could be effectively transferred to the final feature map.

In order to investigate the influences of parameters and structures on the performance of U-Net models in landslide detection, three types of U-Net model—namely, UNet−, UNet+, and ResUNet—were proposed and tested in this study. UNet− has 18 convolutional layers, while UNet+ has 22 convolutional layers. In ResUNet, 23 residual blocks were used to replace the convolutional layers of the original UNet model, similarly to in [31]. 

#### 2.3.5. Performance Assessment

Five typical indicators were used to evaluate the performances of the above models in landslide detection, including the accuracy, precision, recall, and F1 score. Additionally, a statistical significance test was designed to quantify the likelihood scores of two models, with the known F-test. When a very low *p*-value is obtained (*p* < 0.05), there is a statistically significant difference between models. 

In the following formulae of performance indicators, true positive (*TP*) means that both the detected and the reference are landslides; false positive (*FP*) means that the detection is landslide but the reference is not; true negative (*TN*) means that both the detected and the reference are non-landslide; and false negative (*FN*) means that the detected is non-landslide but the reference is landslide.

(1) $$\mathrm{Accuracy}=\frac{TP+TN}{TP+TN+FP+FN}$$

Accuracy is the proportion of correctly detected samples to all samples. It is used to indicate the overall performance of a model.

(2) $$\mathrm{Precision}=\frac{TP}{TP+FP}$$

Precision is the ratio of true positive samples to all detected positive samples. The higher the precision, the greater the probability of the model finding positive samples.

(3) $$\mathrm{Recall}=\frac{TP}{TP+FN}$$

Recall is the proportion of true positive samples to all positive samples in the area. The higher the recall rate, the higher the probability of the model detecting all positive samples. The recall rate reflects the sensitivity of the model to identify positive samples.

(4) $$F1\;\mathrm{Score}=\frac{2\;\cdot \mathrm{Precison}\cdot \mathrm{Recall}}{\mathrm{Precison}+\mathrm{Recall}}$$

F1 Score is the harmonic average value of Precision and Recall. If, and only if, both Precision and Recall are 1, the F1 Score is equal to 1, implying that the model identifies all true positive samples successfully.

## 3. Results

**Figure 7:**
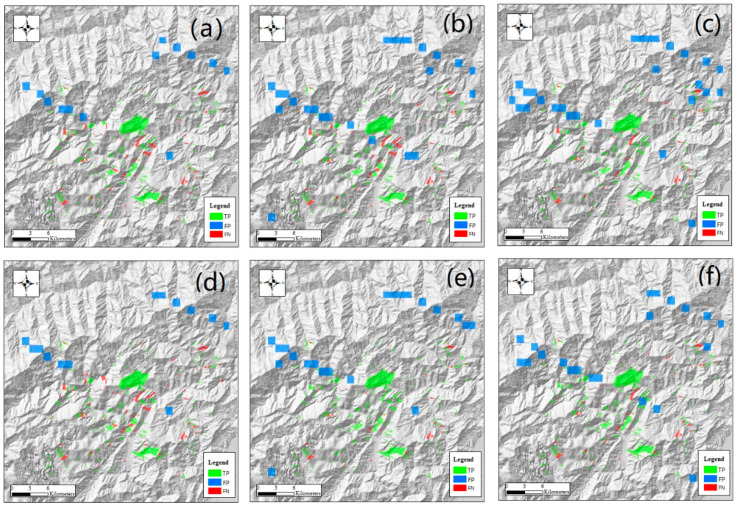
Detection results of scene-oriented models with sample set S10000 around the Daguangbao landslide (the central one) in the Wenchuan earthquake area: (**a**) VGG16; (**b**) ResNet50; (**c**) DenseNet120; (**d**) VGG19; (**e**) ResNet101; (**f**) DenseNet201. The Daguangbao landslide is known for its area (7.11 km^2^) and height difference (1567 m) [46]. Here, landslides correctly detected (*TP*) or missed (*FN*) are shown in the form of polygons, while scenes misidentified as landslides (*FP*) are shown in blue rectangles. The blurred patch in the southwest is a defect of the original DEM, not related to the detection result.

**Figure 8:**
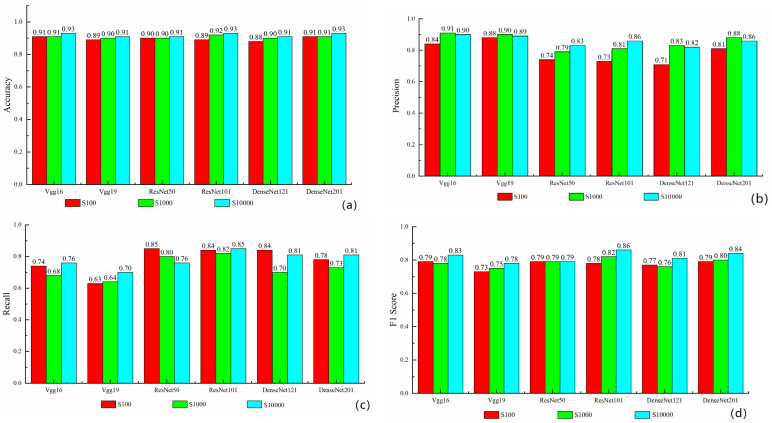
Statistical performance of scene-oriented models: (**a**) accuracy; (**b**) precision; (**c**) recall; (**d**) F1 score.

**Figure 9:**
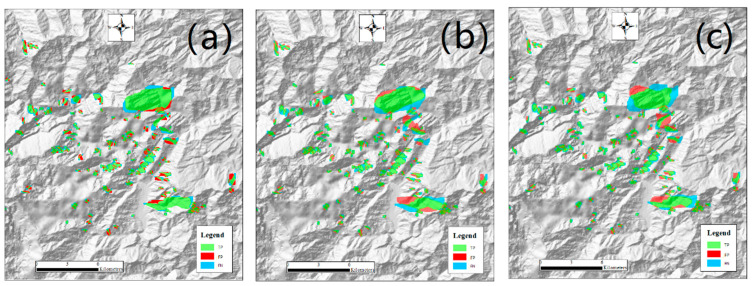
Detection results of U-Net models with sample set S10000 around the Daguangbao landslide: (**a**) UNet−; (**b**) UNet+; (**c**) ResUNet. The blurred part in the southwest is a defect of the original DEM, not related to the detection result.

**Figure 10:**
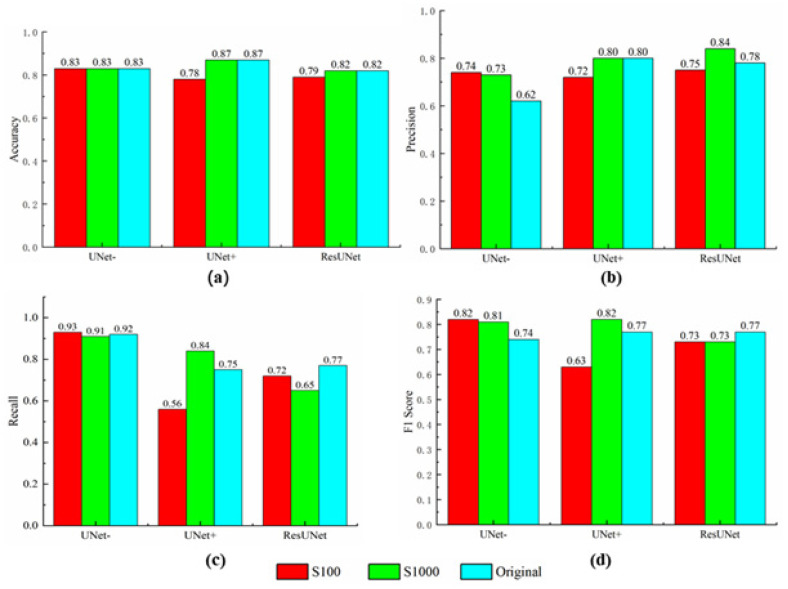
Statistical performance of the UNet, UNet+, and ResUNet models: (**a**) accuracy; (**b**) precision; (**c**) recall; (**d**) F1 score.

In this study, VGG16, VGG19, ResNet50, ResNet101, DenseNet120, DenseNet201, UNet−, UNet+, and ResUNet were trained with S100, S1000, and S10000. The detection results of scene-oriented models within the target area are shown in Figure 7 and Figure 8, and Supplementary Figures S1 and S2. The results of U-Net models are displayed in Figure 9 and Figure 10, and Supplementary Figures S3 and S4. Statistical significance test results are shown Figures S5 and S6. Related confusion matrices are shown in Supplementary Tables S1–S3.

Figure 7 shows that VGG models produced the fewest FP patches, indicating that the fewest scenes are misclassified as landslides in its results. When the VGG model becomes deeper, the number of FP patches reduces. Similarly, ResNet101 and DenseNet201 generate fewer FP patches than their shallower models (ResNet50 and DenseNet120, respectively). Among all panels, the most FPs are found in the DenseNet120 results. It is suggested that deeper models have the better ability to reduce overdetection. The most FNs and the fewest FNs are discovered in the VGG19 and ResNet101 results, respectively. For ResNet, when the number of layers increases, the number of FNs decreases remarkably, i.e., ResNet101 has far fewer FNs than ResNet50. For other models, increasing the number of layers does not lead to an absolute decrease in the number of FNs. 

Supplementary Figures S1 and S2 illustrate detection results with smaller sample sets (S1000 and S100, respectively). It can be observed that the FN and FP numbers of the VGG results did not change much when the number of samples decreased. This implies that VGG could maintain performance well when the number of samples decreases. With regard to the other two models, both the FP and FN numbers barely change when tested with 1000 samples; then, a dramatic increase in the number of FPs and unchanged FNs is witnessed when the number of samples decreases to 100. When only 100 samples are available, many non-landslide scenes are misidentified as landslides, while the number of landslides misidentified as non-landslide does not increase much. It is worth noting that increasing the number of layers of DenseNet could apparently improve the performance of landslide detection, as the number of FPs and FNs in the DenseNet201 results is far fewer than in the DenseNet120 results. Finally, it can be concluded that 1000 samples seem sufficient for model training, as the results from this sample size are similar to those from 10,000 samples—especially for VGG and DenseNet201—while 100 samples are not recommended, because the results from this sample size are much worse. 

It should be noted that the distributions of FPs and FNs in all panels are similar, suggesting that misclassifications often occurred in specific slopes. This can be explained in that for those areas sharing similar spectral and spatial characteristics with landslides (e.g., bare slopes), it is hard to distinguish them no matter which model is used. It can also be observed that greater landslides are more likely to be correctly detected in all models’ results.

The accuracy of all models is about 0.9, and increases along with the growth of the sample size, as shown in Figure 8a. Although this indicator seems very promising, it cannot reflect the real performance of landslide detection, as non-landslide scenes are also involved. In short, high accuracy does not necessarily mean high correctness of landslide detection. 

Figure 8b shows that VGG models achieve the highest precision among all models. The precision is similar when tested with S1000 and S10000, while it decreases a little when the sample size decreases to 100. Figure 8c shows that VGG models obtain the lowest recall rate, which fluctuates a little when the sample size decreases. With regard to ResNet models, the precision is a little lower than that of DenseNet models, which decreases evidently when the sample size reduces. Fortunately, ResNet models obtain the best recall rate among all models, which also fluctuates a little when the sample size decreases. For DenseNet models, the precision also decreases significantly when the sample size reduces to 100, while the difference between tests with S1000 and S10000 is tiny. Unfortunately, the recall rate with S1000 is much lower than those with other sample sets. 

With regard to F1 Score, ResNet101 with S10000 samples achieves the highest one among all tests, followed by DenseNet201 with S10000. For ResNet and DenseNet, the F1 score rises along with the increase in the number of samples or layers. For VGG, the F1 score also increases when the samples grow, but the increased number of layers results in a lower F1 score. Although no model could obtain both the highest precision and the best recall at the same time, it can be concluded that a larger sample size can lead to better results if the F1 score is employed as a comprehensive measure, and the increase in the number of layers could also bring higher performance for ResNet and DenseNet. Given that tens of thousands of samples are often not available in most applications, ResNet101 with 1000 samples is recommended for scene-oriented landslide detection according to the F1 score. 

In Figure 9, the TP, FP, and FN of U-Net models are represented by pixels. In this case, some landslide pixels may be misclassified as non-landslide pixels, but it becomes impossible to miss all pixels of a landslide. From the figure, it can be observed that TP pixels often reside at the central part of a landslide, while FP and FN pixels are more likely to be located at the transition between the landslide and non-landslide cover. For these pixels, their spectral and spatial characteristics are often not distinguishable. Specifically, the most FPs and least FNs are found in the UNet− results (Figure 9a), while the other two models have similar FN and FP numbers. As shown in Supplementary Figures S3 and S4, when the number of samples decreases, the number of FPs increases slightly in UNet−, the number of FNs increases noticeably in UNet+, and both FPs and FNs increase slightly in ResUNet. 

Figure 10a shows that the overall accuracy of all models is about 0.8, and it increases along with the growth in the number of samples. Figure 10 shows that UNet− obtains the worst precision rate and the best recall rate, while UNet+ and ResUNet achieve similar precision and recall rates. It can be observed that the test with 1000 samples obtains good precision for all models, and a satisfactory recall rate for UNet− and UNet+. According to Figure 10d, UNet− with S100 and S1000, and UNet+ with S1000, obtain better F1 scores than other tests. Thus, UNet− is recommended for pixel-based landslide detection, when a large number of samples are not available. Compared with Figure 8, the best precision of U-Net are a little lower than those of DenseNet201 and ResNet101, while the Recall of UNet− is evidently higher than that of all scene-oriented models. Specifically, fewer non-landslides are misidentified as landslides in scene-oriented models, while less landslide pixels are missed by UNet−.

Supplementary Figures S5 and S6 illustrate that when tested with a large number of samples (e.g., 1000 or 10,000 samples, there is no significant difference between models’ performance. When the number of samples decreases, differences between models increase and become significant. In particular, ResNet101 becomes significantly different from other models when 100 samples are used. This suggests that sample size is critical to deep-learning-based landslide detection.

## 4. Discussion

Several typical deep learning models—including VGG, ResNet, DenseNet and U-Net, with varied structures, layers and samples—were involved in the comparative experiments in the Wenchuan earthquake area. We found that most of the performance indicators, except for accuracy, were a little lower than in other experiments [28,29,30,31,32,33,34]. The serious false positives and false negatives may be caused by the large number of bare slopes—ranks that have similar spectral and spatial characteristics to landslides in this high mountain area. Generally, larger sample size leads to better performance for VGG, ResNet, and DenseNet, while this is not the case for UNet. For ResNet and DenseNet, increasing the number of layers could improve their results, while fewer layers achieve higher performance for VGG and UNet models. Considering the cost of generating samples, 1000 samples are suggested for deep-learning-based landslide detection. 

The difference between the scene-oriented models and the pixel-based methods should be noted. For detecting landslides, scene-oriented models try to extract their semantic features from remote sensing images. When the model structure is unreasonable or there are too many convolutional layers, redundant features are extracted, and then cause detection errors. In scene-oriented methods, an FN error means that a landslide is completely missed, while an FP error indicates a non-landslide that is mislabeled as a landslide. Moreover, the scene-oriented detection could just provide a very rough outline (e.g., a rectangle) of a detected landslide. In contrast, in the pixel-based results, it is impossible for the pixels of a true landslide to be barely detected, or for all pixels of a detected landslide to be non-landslide [31]. Furthermore, it can be observed that TP pixels often reside in the central part of a landslide, while FP and FN pixels are likely to appear around the TP pixels. These results are helpful for delineating landslides’ boundaries approximately, and then for further landslide investigation. 

It should be noted that the performance of VGG 19 is often lower than that of VGG 16. As no regularization measure is carried out in VGG—such as weight attenuation, batch normalization, etc.—increasing the convolutional layer may generate a large number of redundant features in the learning process, known as “overfitting” [43]. Furthermore, the large pre-training weight of VGG models may also bring uncertainties to landslide detection. Our test also finds that the huge number of parameters often leads to high computational complexity when training and applying the models, even though only the classifier needs to be trained.

With regard to ResNet, it learns not only the original input data, but also the residuals of the input data, which could alleviate the problem of gradient vanishing [29]. Then, it is possible to improve landslide detection performance by increasing the number of convolutional layers [41]. This was verified in this test, where ResNet101 showed better recall, precision, and F1 score than ResNet50. The study also discovers that when the number of layers increases to more than 1200, the model’s performance becomes similar to ResNet101. That is to say, the performance of ResNet models cannot be improved infinitely by simply increasing the number of convolutional layers, due to the overfitting problem. 

For improving network performance, some models (e.g., Inception series) rely on increasing the width of the network, while ResNet inclines to increase the number of convolutional layers [47]. Unlike these, DenseNet tries to reduce network parameters and alleviate the gradient vanishing problem via feature reuse and bypass settings [26]. In DenseNet, the input of each layer becomes the superposition of all previous layers, and then its feature map is passed to all subsequent network layers. In this study, the parameters of DenseNet201 are much less than ResNet101, while they achieve similar precision, recall, and F1 score. It should be noted that the pre-training weight becomes an important factor affecting their performance. Since the pre-training weights of DenseNet and ResNet were obtained with the same sample set (ImageNet) and strategies, they show similar performances in this test. 

In addition, three UNet models (e.g., UNet−, UNet+, and ResUNet) were proposed and tested for landslides detection. The design and training strategies—such as batch normalization and weight attenuation—were learned from the original U-Net model. Tests indicate that UNet− and UNet+ with 1000 samples obtain higher F1 scores, while the performance of tests with S10000 is evidently lower. This challenges the idea that greater sample size would lead to higher performance. 

## 5. Conclusions

Typical deep learning models—including VGG, ResNet, DenseNet and U-Net—with different structures, layers, and samples, were tested comparatively for landside detection in the Wenchuan earthquake area. Although the number of models, sample sets, and layers involved are limited, subject to time and computational capacity, some interesting results were still found. For VGG, ResNet, and DenseNet, a larger sample set often leads to better performance, while tests with 10,000 samples achieve lower F1 scores for UNet models. Moreover, the increase in the number of layers could improve the detection results for ResNet and DenseNet. If tens of thousands of samples are not available in applications, 1000 samples are recommended for deep-learning-based landslide detection. It should be noted that scene-oriented detection could provide a rough outline of a detected landslide, while pixel-based models are able to delineate landslides’ boundaries approximately.

Based on tests with a large number of samples, this study provides valuable clues for designing models’ type, layers, and sample size, and suggests that what matters to a project (e.g., *TP*, *FN*, *FP*, cost, time, or landslide’s boundaries) should be investigated first. 

## Data Availability

The data presented in this study are openly available in the landslide inventories: https://www.sciencebase.gov/catalog/item/583f4114e4b04fc80e3c4a1a (accessed on 17 January 2021); the image website: http://www.gscloud.cn/sources/index?pid=2&rootid=2 (accessed on 17 January 2021), https://www.arcgis.com/index.html (accessed on 17 January 2021).

## Supplementary Materials

The following are available online at https://www.mdpi.com/article/10.3390/s21155191/s1, Figure S1: Detection results of scene-oriented models with sample set S1000 around the Daguangbao landslide; Figure S2: Detection results of scene-oriented models with sample set S100 around the Daguangbao landslide; Figure S3: Detection results of U-Net models with sample set S1000 around the Daguangbao landslide; Figure S4: Detection results of U-Net models with sample set S100 around the Daguangbao landslide. Figure S5: Statistical significance test of scene-oriented models; Figure S6: Statistical significance test of UNet models.
