# Supplementary material for: Valuable Clues for DCNN-Based Landslide Detection from a Comparative Assessment in the Wenchuan Earthquake Area
Source: Sensors (Basel). 2021 Jul 31;21(15):5191. doi: 10.3390/s21155191 (PMC8348725; doi:10.3390/s21155191)
Supplement: Supplementary file 1 [file sensors-21-05191-s001.zip › sensors-1298556-supplementary.pdf]

Table S1. Confusion matrix of tests with S10000 in the target area*

	**VGG16**	**VGG19**	**ResNet50**	**ResNet101**	**DenseNet120**	**DenseNet201**	**UNet-**	**UNet+**	**ResUNet**
TP	151	139	151	170	161	163	92	264	242
FP	16	17	30	27	36	27	57	73	62
TN	655	654	640	644	635	644	713	452	487
FN	48	59	48	30	38	37	8	81	79

*All landslide and non-landslide samples in the target area are involved. The number of latter is much more than that of the former.

Table S2. Confusion matrix of tests with S1000 in the target area

	**VGG16**	**VGG19**	**ResNet50**	**ResNet101**	**DenseNet120**	**DenseNet201**	**UNet-**	**UNet+**	**ResUNet**
TP	156	128	159	164	141	145	323	259	218
FP	14	15	43	37	28	19	118	64	42
TN	657	656	629	633	643	652	396	497	496
FN	64	71	39	35	58	54	33	50	115

Table S3. Confusion matrix of tests with S100 in the target area

	**VGG16**	**VGG19**	**ResNet50**	**ResNet101**	**DenseNet120**	**DenseNet201**	**UNet-**	**UNet+**	**ResUNet**
TP	170	125	171	168	168	155	326	164	249
FP	28	17	59	61	70	37	117	64	82
TN	643	653	612	610	601	634	401	517	441
FN	51	74	29	31	31	44	26	126	98

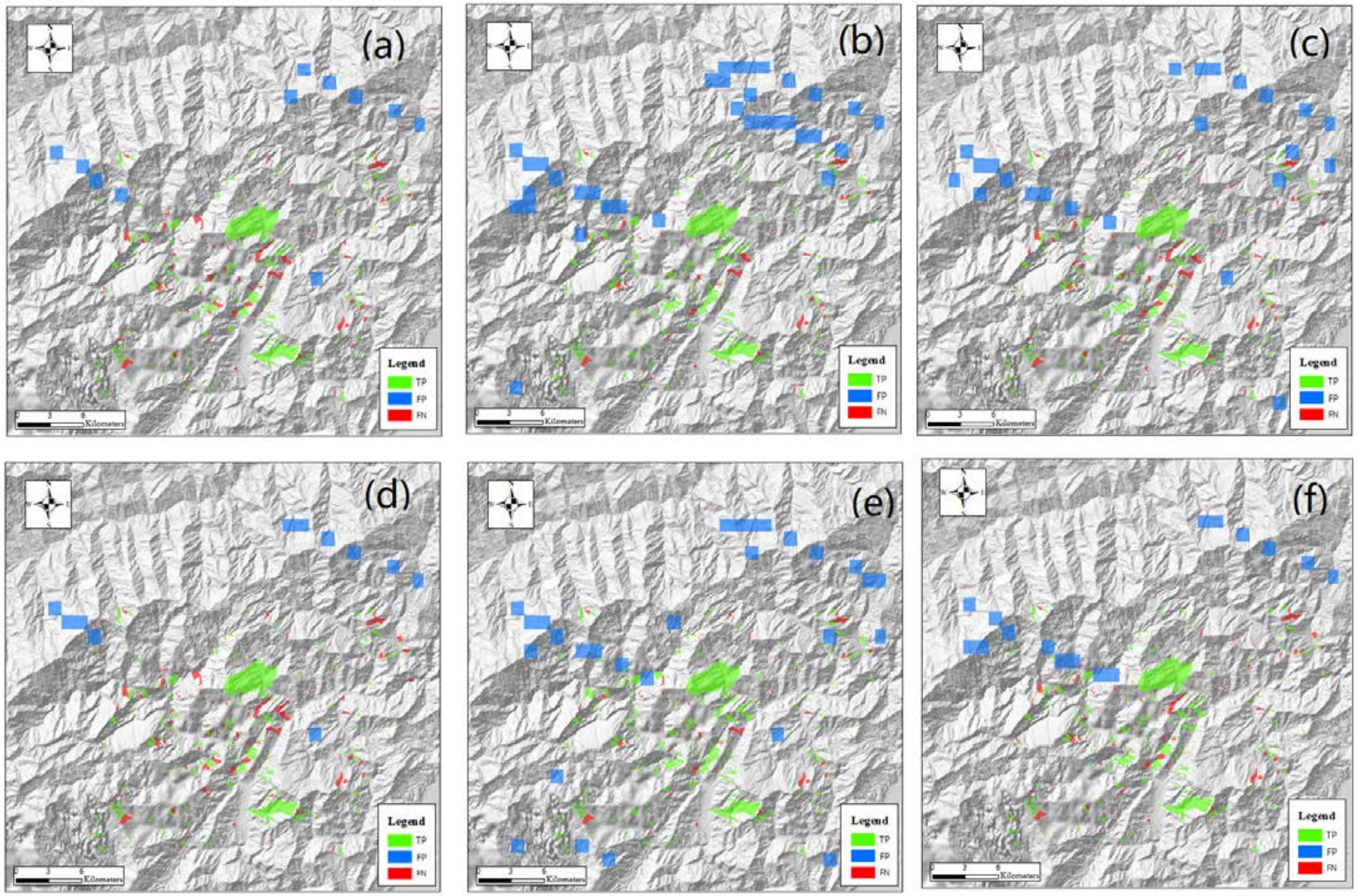

Figure S1. Detection results of scene-based models with sample set S1000 around the Daguangbao landslide. (a) VGG16; (b) ResNet50; (c) DenseNet120; (d) VGG19; (e) ResNet101; (f) DenseNet201.

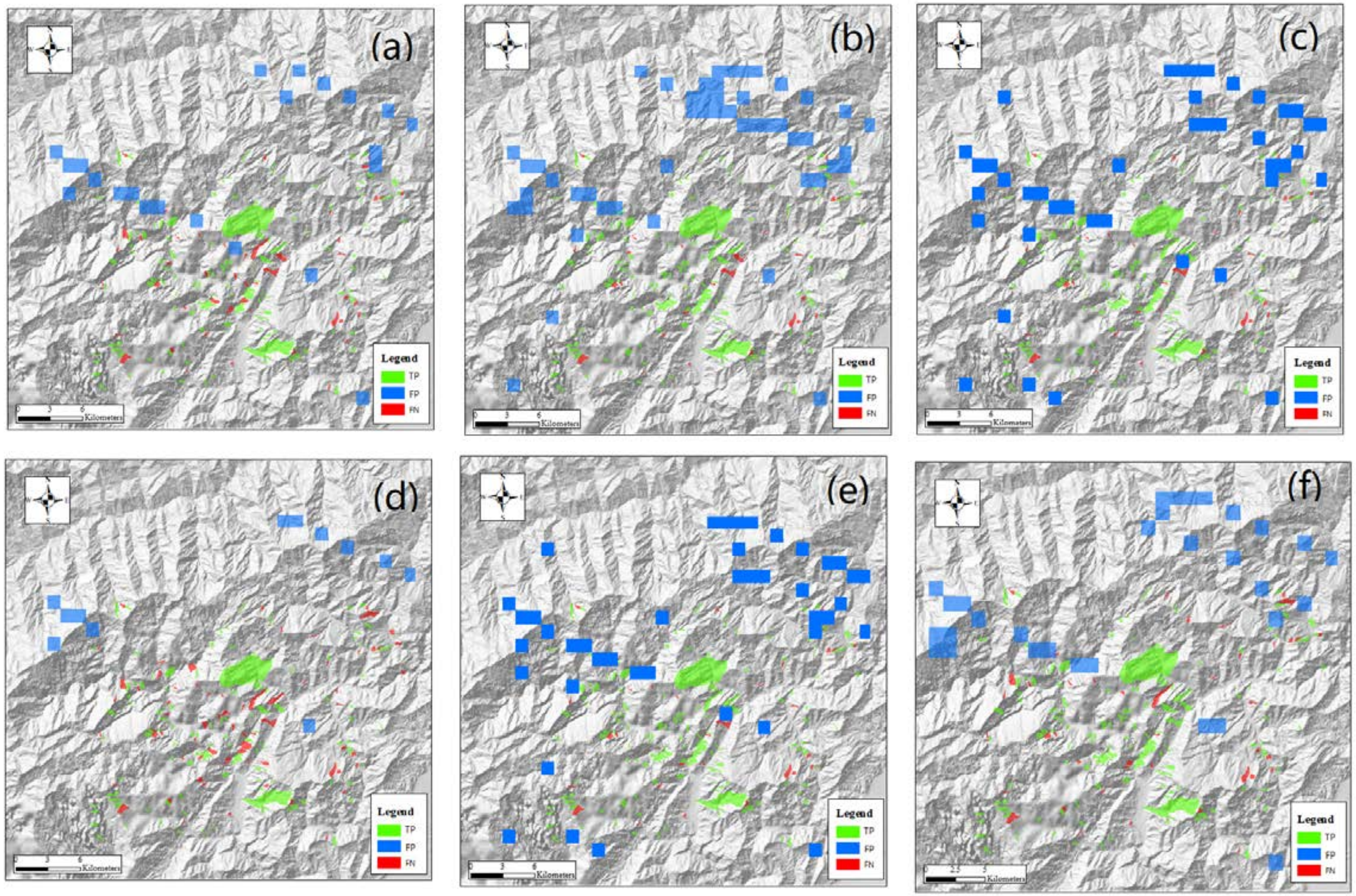

Figure S2. Detection results of scene-based models with sample set S100 around the Daguangbao landslide. (a) VGG16; (b) ResNet50; (c) DenseNet120; (d) VGG19; (e) ResNet101; (f) DenseNet201.

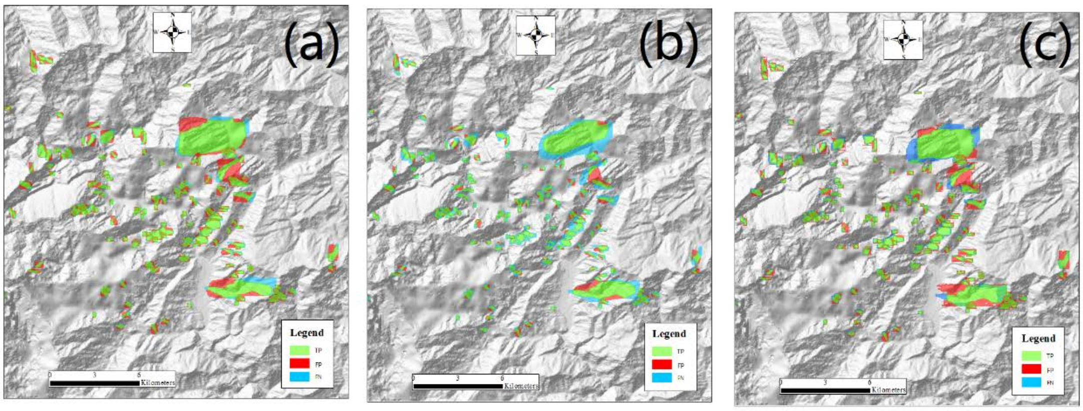

Figure S3. Detection results of U-Net models with sample set S1000 around the Daguangbao landslide. (a) UNet-; (b) UNet+; (c) ResUNet.

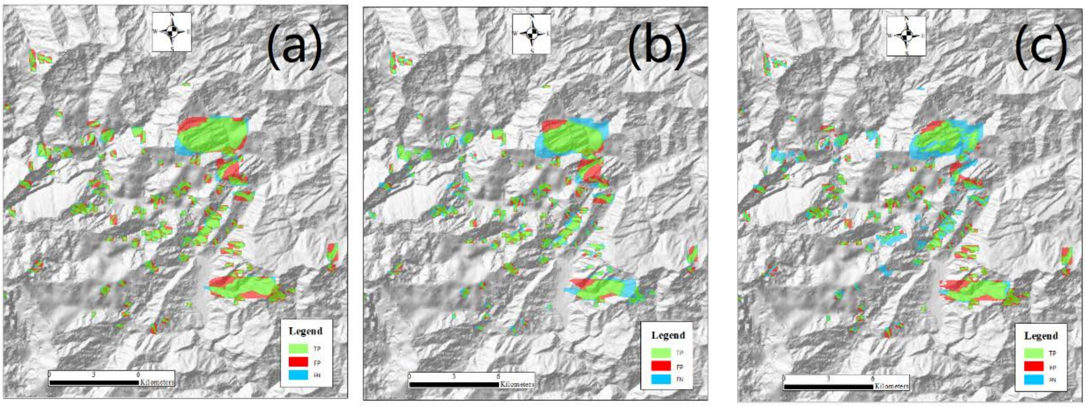

Figure S4. Detection results of U-Net models with sample set S100 around the Daguangbao landslide. (a) UNet-; (b) UNet+; (c) ResUNet.

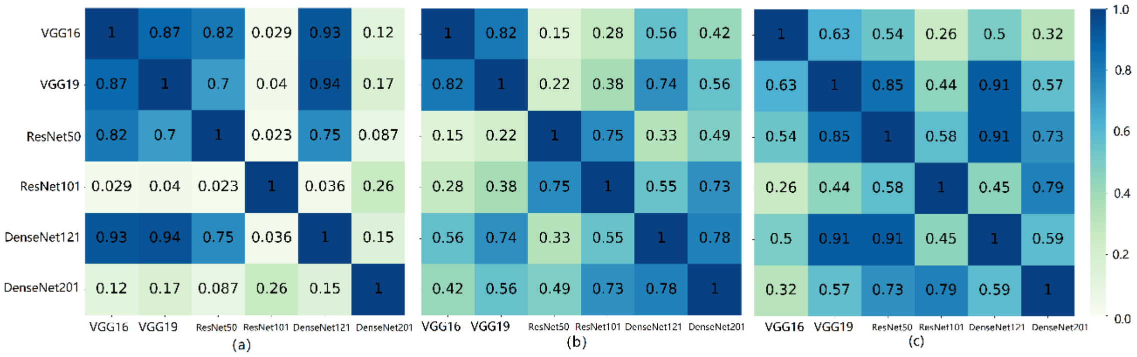

Figure S5. Statistical significance test of scene-oriented models. (a) test with 100 samples; (b) test with 1000 samples; (c) test with 10,000 samples. The data in cells is the p-value of F-test.

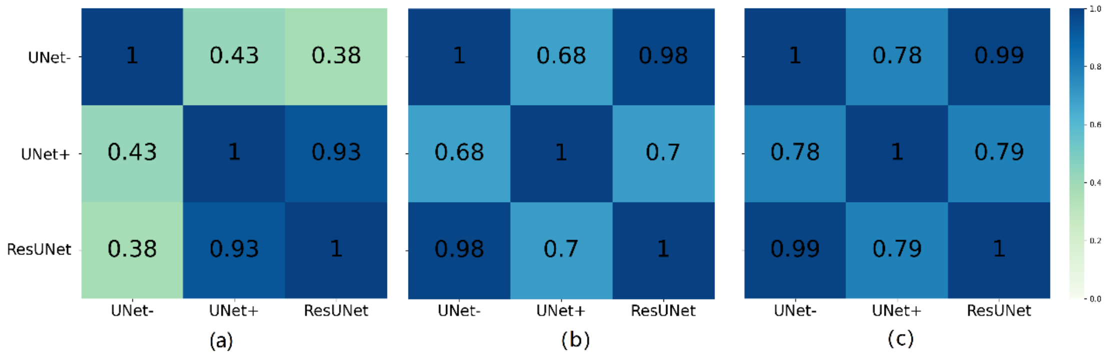

Figure S6. Statistical significance test of UNet models. (a) test with 100 samples; (b) test with 1000 samples; (c) test with 10,000 samples. The data in cells is the p-value of F-test.

Open access resources

The landslide inventories
https://www.sciencebase.gov/catalog/item/583f4114e4b04fc80e3c4a1a
the image website
http://www.gscloud.cn/sources/index?pid=2&rootid=2
